# Long-term safety and efficacy of Omnitrope^®^ in adults with growth hormone deficiency: Italian interim analysis of the PATRO Adults study

**DOI:** 10.1007/s40618-016-0604-8

**Published:** 2017-02-04

**Authors:** D. Ferone, E. Profka, V. Gasco, M. R. Ambrosio, A. Colao, C. Di Somma, E. Puxeddu, G. Arnaldi, C. Pagano, E. Zecchi, A. Pietropoli, P. Beck-Peccoz

**Affiliations:** 1 Endocrinology Unit, Department of Internal Medicine and Medical Specialties (DiMI), Center of Excellence for Biomedical Research (CEBR), IRCCS AOU San Martino-IST, University of Genova, Viale Benedetto XV, 6, 16132 Genova, Italy; 2 Endocrinology and Metabolic Diseases Unit, Department of Clinical Sciences and Community Health, Fondazione Istituto di Ricovero e Cura a Carattere Scientifico Ca’ Granda Ospedale Maggiore Policlinico, University of Milano, Milan, Italy; 3 Endocrinology, Diabetes and Metabolism, Department of Medical Science, Città della Salute e della Scienza di Torino, University of Torino, Turin, Italy; 4 Section of Endocrinology and Internal Medicine, Department of Medical Sciences, University of Ferrara, Ferrara, Italy; 5 Department of Clinical Medicine and Surgery, University Federico II of Napoli, Naples, Italy; 6 Institute of Diagnostic and Nuclear Research, Fondazione Istituto di Ricovero e Cura a Carattere Scientifico SDN, Naples, Italy; 7 Veterinary and Forensic Biotechnological Sciences Department of Medicine Section, Internal Medicine, Endocrine and Metabolic Sciences, University of Perugia, Perugia, Italy; 8 Clinica di Endocrinologia e Malattie del Metabolismo, Ospedali Riuniti di Ancona, Ancona, Italy; 9 Department of Medicine, Internal Medicine 3, University of Padova, Padua, Italy; 10 Sandoz S.p.A., Origgio, VA Italy; 11 Hexal AG, Holzkirchen, Germany; 12 Endocrinology and Diabetology Unit, Medical Sciences Department, Fondazione Istituto di Ricovero e Cura a Carattere Scientifico Cà Granda Ospedale Maggiore Policlinico, Milan, Italy

**Keywords:** Growth hormone deficiency, Hypopituitarism, Omnitrope®, Recombinant human growth hormone, Insulin-like growth factor-1, Safety

## Abstract

**Purpose:**

To report the long-term effectiveness and safety of the recombinant human growth hormone Omnitrope^®^, a somatropin biosimilar to Genotropin^®^, in Italian patients with growth hormone deficiency (GHD) enrolled in the PATRO Adults study.

**Methods:**

The PATRO Adults study is an ongoing observational, longitudinal, non-interventional global post-marketing surveillance study, conducted in several European countries. The primary endpoint is long-term safety; secondary endpoints include the effectiveness of Omnitrope^®^, which was assessed using serum insulin-like growth factor-1 levels, body composition, bone mineral density and lipid levels. Here we report the data from the Italian patients enrolled in the study.

**Results:**

Sixty-seven patients (mean age 50.4 years, 61.2% male) have been enrolled and have received a mean 45.4 ± 24.3 months of Omnitrope^®^. A total of 55.2% of patients were reported to have experienced adverse events (AEs), including arthralgia, myalgia, abdominal distension and hypoaesthesia, and 4.5% had adverse drug reactions. Fourteen serious AEs have been recorded; none of these are considered related to the study drug. The effectiveness of Omnitrope^®^ was similar to other available somatropin preparations.

**Conclusions:**

This study confirms the effectiveness and safety of Omnitrope^®^ in adult patients with GHD in Italy. However, due to the limited size of the study population, these results need to be further confirmed by the global PATRO Adults study.

## Introduction

Adult-onset growth hormone deficiency (GHD) is a condition associated with abnormal substrate metabolism, body composition, and physical and psychosocial function, due to the inability of the pituitary gland to produce enough growth hormone (GH) [1]. Consequently, patients with adult-onset GHD have difficulties in controlling their body weight, show increased body fat (more specifically abdominal fat), and decreased muscle mass with reduced muscle strength and exercise capacity. Increased anxiety levels and depressed mood also contribute to a reduced quality of life in these patients. Adult-onset GHD can result from hypothalamic-pituitary disease, neurosurgery and/or cranial irradiation during the treatment of pituitary and brain tumours, or traumatic brain injuries [2].

Guidelines for the diagnosis and treatment of adults with GHD recommend replacement therapy with recombinant human GH (rhGH), which is dosed based on plasma insulin-like growth factor-1 (IGF-1) levels and the presence or absence of side effects [1, 3]. Replacement therapy has been shown to correct the metabolic, functional and psychological abnormalities associated with adult GHD [4]. Lipolysis is increased in response to rhGH replacement therapy, resulting in a reduction of total body (mainly visceral) fat, while one year of rhGH therapy seems to reduce early cardiac organ damage in adult patients with GHD [5]. It has also been suggested that exercise capacity and physical performance can be improved by this treatment [1, 3].

One rhGH available for the treatment of GHD in adults is Omnitrope^®^ (Sandoz, Kundl, Austria). Omnitrope^®^, expressed by a transformed strain of *Escherichia coli*, is biosimilar to Genotropin® (Pfizer Limited, Sandwich, UK) and was the first product to be approved by the European Medicines Agency (EMA) in 2006 using the European biosimilar regulatory pathway [6].

In adults, Omnitrope^®^ is approved for the replacement of adult- or childhood-onset GHD. Studies in adult patients suggest that switching from Genotropin^®^ to Omnitrope^®^ has no impact on the safety and efficacy profile [7, 8]; however, data assessing the long-term safety and effectiveness of Omnitrope^®^ in adults in the setting of routine clinical practice are not yet available. To address this, the PAtients TReated with Omnitrope^®^ (PATRO) Adults study was initiated as part of the Omnitrope® Active Pharmacovigilance Program agreed with the EMA upon the approval of Omnitrope^®^. PATRO Adults is a long-term post-marketing surveillance (PMS) study investigating the long-term safety and effectiveness of Omnitrope^®^ in adults with GHD. It is being conducted as part of the Risk Management Plan for Omnitrope^®^, to fulfil the commitment with the EMA [9]. Preliminary results of this study indicate that Omnitrope^®^ is well tolerated in routine clinical practice [10–12]. Between September 2007 and September 2015, 954 patients from eight different countries (Czech Republic, France, Germany, Italy, Spain, Sweden, The Netherlands, and UK) were enrolled in the PATRO Adults study. Here we report the results of all patients enrolled in Italy.

## Materials and methods

### Study design

PATRO Adults is a multicentre, open-label, longitudinal, non-interventional PMS study, which was initiated in September 2007 and is being conducted in hospitals and specialised endocrinology centres in various countries where Omnitrope^®^ is approved and regularly prescribed. The design and methods of this study have been published in detail previously [9]. Briefly, eligible patients are adults (≥15 years old) receiving treatment with Omnitrope® for GHD (isolated or combined with other hormone deficiencies) and who provided written informed consent. Patients who had received a previous rhGH were also eligible for inclusion. The study was reviewed and approved by each study site’s Independent Ethics Committee or Institutional Review Board before the starting and was conducted in accordance with the Declaration of Helsinki.

### Treatment and outcomes

Patients included in the PATRO Adults study received Omnitrope^®^ treatment in accordance with the recommendations in the Summary of Product Characteristics [13] and/or the prescribing information of the respective countries. The primary objective of this PMS study was to collect and analyse data on the long-term safety of Omnitrope^®^ in adults treated within routine clinical practice to extend the global safety database of Omnitrope^®^. Aspects of clinical safety, such as risk of developing glucose intolerance or diabetes and occurrence of malignancies, were examined along with the effects on cardiovascular risk factors, such as blood pressure and inflammatory markers. All adverse events (AEs), serious adverse events (SAEs), adverse drug reactions (ADRs), and serious adverse drug reactions (SADRs) were collected and recorded in electronic case report forms (e-CRF) and entered into the Sandoz Safety Database.

The secondary objective was to monitor the treated population and to collect data on effectiveness issues related to Omnitrope^®^ treatment. These included measurements of IGF-1 levels within age-and gender-adjusted normal ranges, and the fasting lipid profile. Body composition assessment was carried out using anthropometric measures (weight, waist and hip circumferences, total fat mass, lean body mass and body mass index [BMI]). All evaluations and assessments were performed at each local centre, according to the local practice.

### Statistical analysis

The safety analysis set (SAF) included all patients who had documentation of any data in an e-CRF whilst the effectiveness analysis set (EFF) included all patients who had at least one dose of Omnitrope^®^. Statistical analyses for this study were performed using the software package SAS (version 9.3).

All AEs were coded using MedDRA version 17.1. Concomitant medication was coded according to WHO Drug Dictionary (version 14.3) and the medications were tabulated by Anatomical Therapeutic Chemical term in their current version. For continuous/quantitative variables, descriptive statistics including the number of data values available, number of data values missing, arithmetic mean, standard deviation, minimum, median and maximum were calculated. When appropriate, continuous parameters were compared using *t* tests or Wilcoxon non-parametric tests. For categorical/qualitative variables, frequency and percentage tables were generated. When appropriate, categorical data were compared using Chi-square or Fisher exact tests. Statistical tests were two sided at the significance level of 0.05.

## Results

As of August 2015, 67 patients (mean age 50.4 years, 61.2% male) had been enrolled from eight sites in Italy (Table 1) and received a mean 45.4 ± 24.3 months of Omnitrope^®^ treatment. Of these, 15 patients (22.4%) had discontinued treatment at the time of analysis; reasons for discontinuation included: patients did not wish to continue injections (*n* = 2; 13.3%), AEs (*n* = 1; 6.7%), referral to another endocrinologist (*n* = 1; 6.7%), non-compliance (*n* = 1; 6.7%) and loss to follow-up (*n* = 2; 13.3%). The reason for discontinuation of Omnitrope^®^ was unknown in the remaining eight patients (53.3% of discontinued patients). Overall, 31.3% of patients had received another rhGH before Omnitrope^®^. The mean duration of rhGH pre-treatment was 7.45 ± 4.97 (range 1.6–17.3) years.

**Table 1:** Baseline characteristics and demographics of Italian patients enrolled in the PATRO Adults study as of August 2015

Characteristic	*N* = 67
Gender, *n* (%)	
Male	41 (61.2)
Female	26 (38.8)
Age, years	50.4 ± 14.5
Age group, *n* (%)	
<25 years	4 (6.0)
25–65 years	56 (83.6)
>65 years	7 (10.4)
BMI, kg/m^2^	28.9 ± 5.6
Hip circumference, cm	104.5 ± 18.3
Waist circumference, cm	94.6 ± 14.4
Diagnosis at presentation, *n* (%)	
Isolated GHD	9 (13.4)
Combined GHD	58 (86.6)
Onset of puberty, *n* (%)^a^	
Normal	41 (61.2)
Late	9 (13.4)
Onset of GHD	
Childhood onset	8 (11.9)
Adulthood onset	59 (88.1)
Family history of GHD, *n* (%)	
No	43 (64.2)
Unknown	24 (35.8)
Family history of diabetes, *n* (%)	
No	42 (62.7)
Yes	9 (13.4)
Unknown	16 (23.9)
Family history of autoimmune disease, *n* (%)	
No	44 (65.7)
Yes	2 (3.0)
Unknown	21 (31.3)
Family history of other relevant disease, *n* (%)	
No	30 (44.8)
Yes	22 (32.8)
Unknown	15 (22.4)
Previous treatment status, *n* (%)	
Treatment naïve	46 (68.7)
Pre-treated	21 (31.3)
Concomitant medication for combined GHD patients	
Levothyroxine sodium	51 (87.9)
Cortisone acetate	40 (69.0)
Colecalciferol	28 (48.3)
Testosterone	19 (32.8)
Desmopressin acetate	15 (25.9)
Testosterone undecanoate	13 (22.4)
Acetylsalicylic acid	9 (15.5)
Hydrocortisone	7 (12.1)
Simvastatin	7 (12.1)
Omnitrope^®^ dosing at baseline, mg/kg/day	0.22 ± 0.11
Duration of Omnitrope^®^ treatment, months	45.4 ± 24.3

All values are presented as mean ± standard deviation unless otherwise stated

*BMI* body mass index, *GHD* growth hormone deficiency, *SD* standard deviation

^a^Normal onset of puberty was considered 8–13 years of age in girls and 9–14 years of age in boys; late onset of puberty was considered >13 years of age in girls and >14 years of age in boys

### Safety

As of August 2015, in the current Italian analysis, 89 AEs had been reported in 37 (55.2%) of the 67 patients included in the SAF (Table 2). The most common AEs reported were arthralgia, asthenia and insomnia. A total of six suspected ADRs were reported in three (4.5%) patients and included two arthralgia, two myalgia events, one event of abdominal distension and one of hypoaesthesia. A total of 14 SAEs in 10 (14.9%) patients were reported; however, none were suspected to be related to the study drug. One patient developed a basal cell carcinoma after 2 years of rhGH replacement therapy, but this was not considered drug related and was completely cured. This was the only malignancy reported during the study.

**Table 2:** Adverse events in the SAF (*n* = 67)

	Events, *n*	Patients, *n* (%)
Any AE	89	37 (55.2)
Relationship to study drug		
Not suspected	83	35 (52.2)
Intensity		
Mild	54	33 (49.3)
Moderate	32	16 (23.9)
Severe	3	3 (4.5)
Outcome		
Resolved completely	40	22 (32.8)
Resolved with sequelae	3	3 (4.5)
Ongoing	46	25 (37.3)
Medication given		
No	67	30 (44.8)
Yes	21	17 (25.4)
Missing	1	1 (1.5)
Changes to Omnitrope^®^		
Not changed	76	33 (49.3)
Reduced	3	2 (3.0)
Interrupted	8	5 (7.5)
Missing	2	1 (1.5)
SAE	14	10 (14.9)
AEs in ≥2 patients, *n* (%)		
Arthralgia	–	6 (9.0)
Asthenia	–	5 (7.5)
Insomnia	–	4 (6.0)
Headache	–	3 (4.5)
Back pain	–	2 (3.0)
Hypokalaemia	–	2 (3.0)
Muscle spasms	–	2 (3.0)
Myalgia	–	2 (3.0)
Osteopenia	–	2 (3.0)
Paraesthesia	–	2 (3.0)
Weight increased	–	2 (3.0)

*AEs* adverse events, *SAE* serious adverse event

**Fig. 1:**
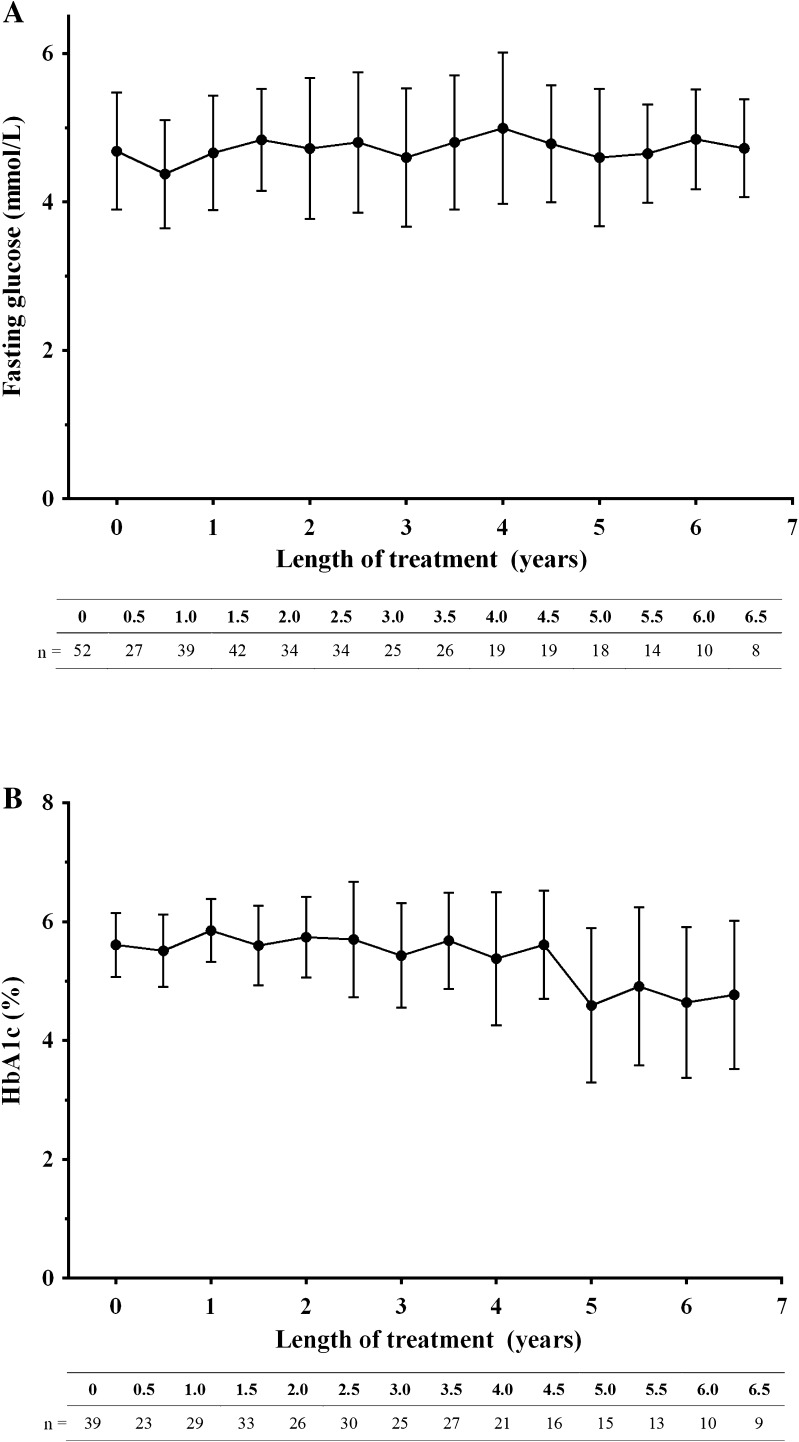
Change in **a** fasting glucose and **b** glycosylated haemoglobin (HbA1c) in the total safety analysis set over the study period

Two patients developed diabetes mellitus during the course of the treatment; however, this was not considered a drug-related event. The mean level of glucose (Fig. 1a) and glycosylated haemoglobin (HbA1c; Fig. 1b) remained unchanged throughout the study period.

### Effectiveness

Body composition changed with Omnitrope^®^ treatment, with slight increases seen in most of the anthropometric parameters investigated over the treatment period; however, none of these were significant (Table 3).

**Table 3:** Change in body composition in the EFF

Anthropometric parameter	Waist circumference, cm		Lean body mass		Total fat mass		Weight, kg		BMI, kg/m^2^	
	*N*	Mean ± SD	*N*	Mean ± SD	*N*	Mean ± SD	*N*	Mean ± SD	*N*	Mean ± SD
Baseline	18	94.6 ± 14.4	9	57.55 ± 7.03	10	35.3 ± 9.4	46	80.4 ± 19.0	46	28.8 ± 5.7
0.5 years	7	98.8 ± 24.3	1	–	1	–	18	80.7 ± 23.5	15	27.9 ± 5.9
1.0 years	17	94.5 ± 15.4	5	63.9 ± 8.6	5	35.4 ± 7.5	36	78.1 ± 18.5	31	28.9 ± 5.4
1.5 years	14	97.8 ± 13.7	5	59.6 ± 5.8	7	36.1 ± 7.2	31	82.20 ± 23.1	25	29.0 ± 6.3
2.0 years	9	95.7 ± 9.4	0	–	0	–	30	76.9 ± 19.1	22	27.0 ± 4.4
2.5 years	6	89.7 ± 10.0	0	–	0	–	22	79.2 ± 18.3	17	28.1 ± 4.3
3.0 years	5	90.2 ± 9.7	1	–	2	40.9 ± 0.3	20	72.5 ± 11.7	14	26.6 ± 4.2
3.5 years	10	97.9 ± 14.1	3	65.2 ± 1.3	6	37.8 ± 6.3	18	76.9 ± 16.5	15	27.9 ± 4.0
4.0 years	6	105.5 ± 18.5	1	–	1	–	22	77.2 ± 16.2	21	27.5 ± 3.9
4.5 years	2	90.5 ± 2.1	0	–	0	–	15	72.4 ± 15.4	13	27.0 ± 4.7
5.0 years	4	94.5 ± 10.7	2	45.4 ± 34.5	3	39.1 ± 7.8	16	75.2 ± 11.7	14	27.3 ± 4.1
5.5 years	3	96.2 ± 5.8	1	–	4	37.6 ± 6.4	9	72.6 ± 12.6	8	27.2 ± 2.3
6.0 years	2	88.3 ± 4.6	1	–	2	38.5 ± 1.6	10	81.2 ± 13.5	9	29.3 ± 5.2
6.5 years	1	–	0	–	0	–	7	88.5 ± 12.0	7	26.2 ± 3.7
7.0 years	0	–	0	–	0	–	2	74.0 ± 14.6	2	27.7 ± 4.2

*BMI* body mass index, *N* number of patients

**Fig. 2:**
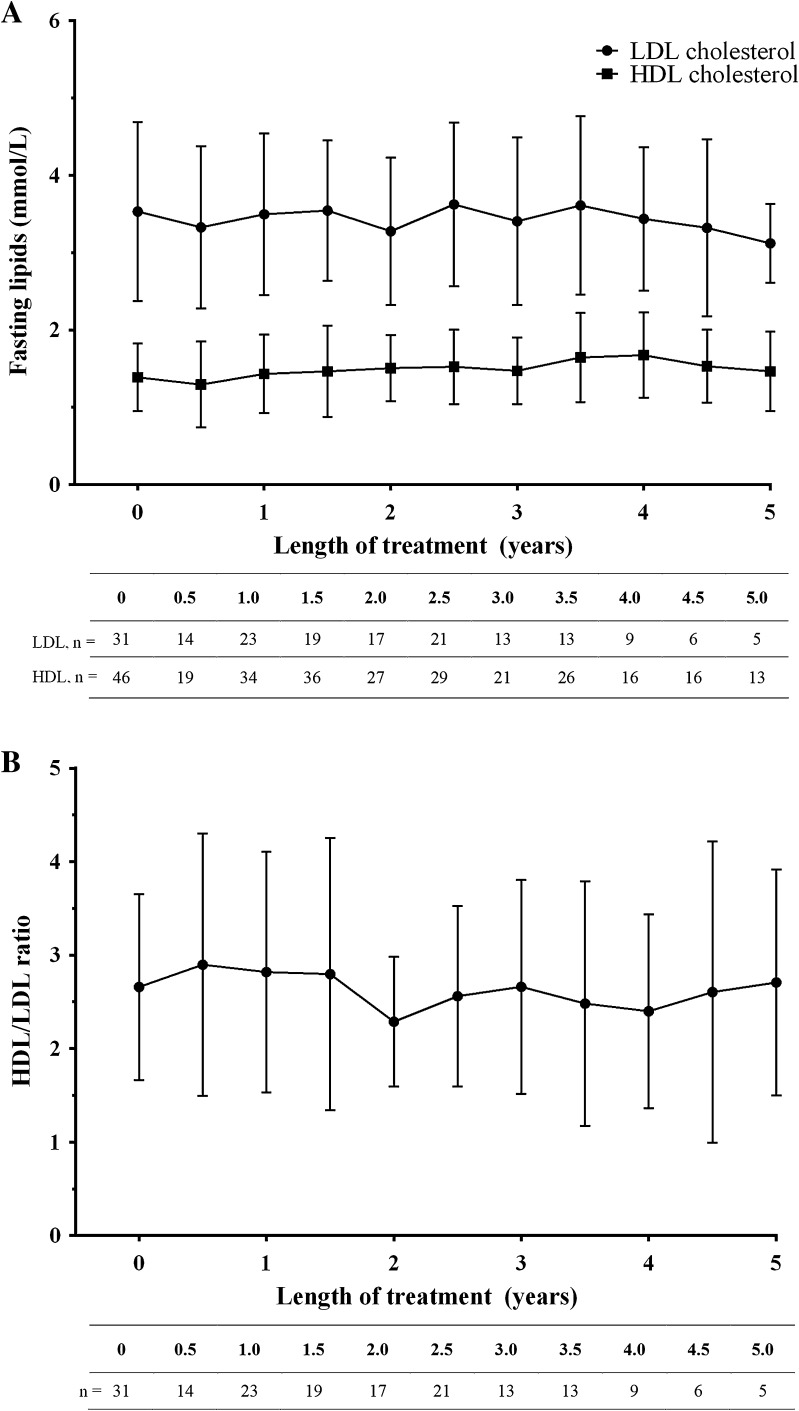
Change in **a** fasting high-density lipoprotein (HDL) cholesterol and fasting low-density lipoprotein (LDL) cholesterol and **b** the ratio of HDL cholesterol and LDL cholesterol in the total safety analysis set over the study period

There was no apparent change in lipid levels over the study duration. A slight decrease in low-density lipoprotein (LDL) cholesterol levels was observed over the duration of the study (Fig. 2a), whereas the ratio of high-density lipoprotein (HDL)/LDL cholesterol over time did not significantly change (Fig. 2b).

**Fig. 3:**
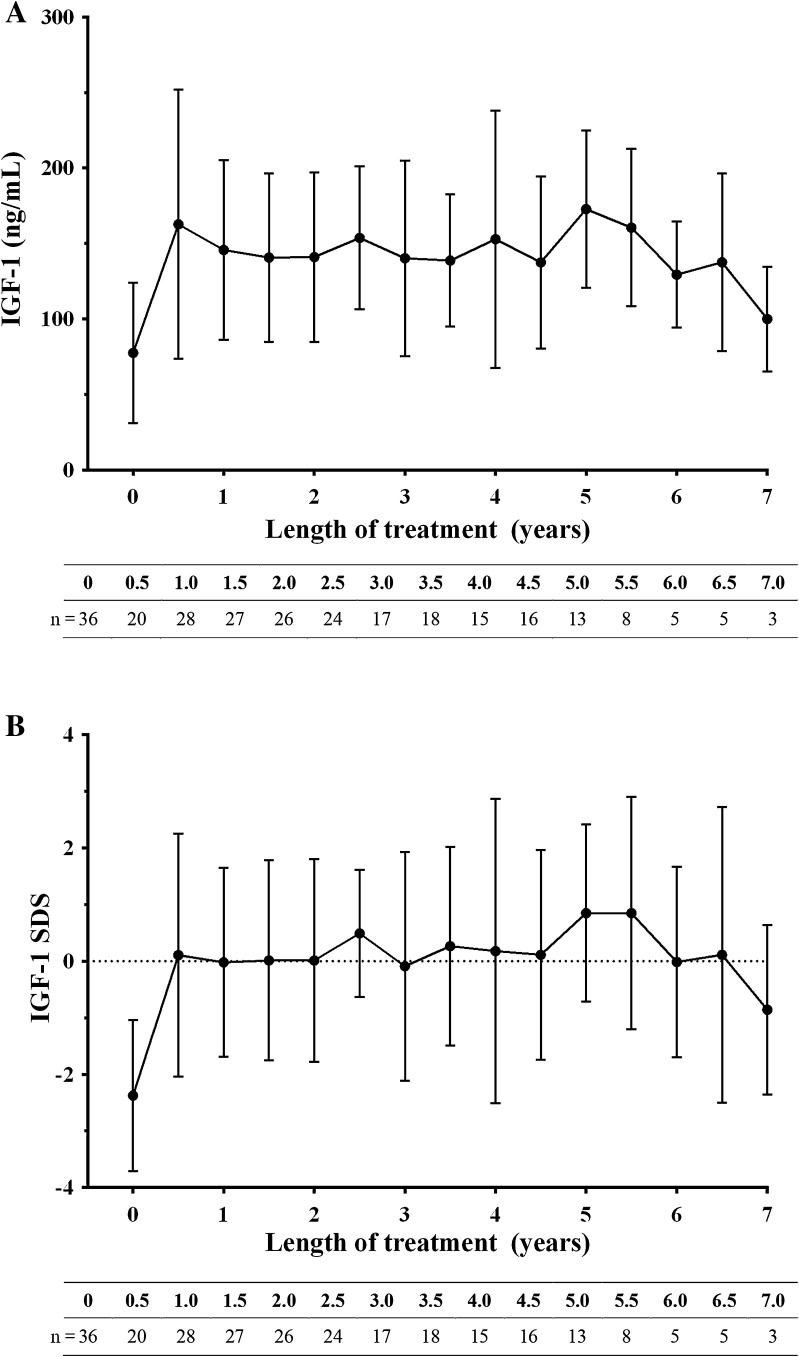
Change in **a** insulin-like growth factor-1 (IGF-1) and **b** IGF-1 standard deviation scores (IGF-1 SDS) in treatment naïve patients included in the efficacy analysis set over the study period

In the first 6 months of Omnitrope^®^ therapy, IGF-1 values increased significantly from baseline in patients who had not received a previous rhGH (Fig. 3). Over the remainder of the study, IGF-1 values remained relatively stable (Fig. 3).

## Discussion

The results of this interim analysis show that Omnitrope^®^ is well tolerated in Italian adults with GHD. The most common AEs reported were arthralgia, asthenia and insomnia, and SAEs were reported in 14.9% of patients. These safety data are consistent with the safety profile of Omnitrope^®^ observed in all patients included in the PATRO Adults study, which has been presented at international conferences in 2013, 2014 and 2015 [10–12]. These reports indicate that Omnitrope^®^ was well tolerated in real-life practice with no confirmed cases of drug-related diabetes, reports of malignancy or other safety issues. The safety profile of Omnitrope^®^ is similar in both adults naïve to rhGH and patients previously treated with other rhGH formulations.

The link between patients receiving replacement rhGH therapy and the possibility of developing diabetes has been a concern, with anecdotal reports of a cause and effect relationship. The incidence of metabolic syndrome is higher in patients with GHD compared with reference populations [14, 15]. However, there are conflicting reports regarding the effect of GH replacement on glucose metabolism. Some studies have shown that rhGH treatment may accelerate the onset of diabetes in predisposed paediatric individuals and patients receiving rhGH therapy may have an increased incidence of metabolic abnormalities in the long term [16–19]. Conversely, other authors have found no evidence to support an increase in the onset of diabetes mellitus in rhGH-treated patients [16, 20]. The results emerging from this analysis seem to confirm that no link exists between adequate rhGH replacement therapy in adults with GHD and the development of diabetes. In fact, no significant changes in glucose metabolism were recorded in the entire cohort of patients, while in the two cases that developed diabetes mellitus during the study, this was not considered drug-related.

Patients with GHD tend to have increased LDL and decreased HDL cholesterol levels, with both features considered characteristic of metabolic syndrome [3]. However, some studies have suggested that rhGH replacement therapy may reverse this trend, increasing the level of HDL and decreasing the level of LDL [3]. Although the sample size is too small to be conclusive, in the Italian GHD population evaluated in the present study, there was a slight decrease in the level of LDL during treatment with Omnitrope^®^.

As both GH and IGF-1 have mitogenic properties, there is concern that rhGH therapy could increase the risk of developing malignant diseases in treated patients. So far, there is no evidence from the Italian GHD patients included in the PATRO Adults study that rhGH therapy increases the risk of malignancy as only one case was reported, which was not thought to be drug related. These results are consistent with several other studies, which also suggest that adequate replacement therapy with rhGH does not increase the risk of de novo or recurring tumours [21–25].

These results are also in line with other studies which explored the efficacy of Omnitrope^®^ [7, 8]. While the results of this analysis are supportive of those from the long-term interventional trials of rhGH for the treatment of adults with GHD, there are some limitations in the interpretation of these findings. The number of patients included in this interim analysis over the long term was small and unselected, which may bias the results. Nonetheless, despite these limitations we believe that this study is reflective of real-world clinical practice in Italy and may be interpreted as such.

It is of interest to compare our findings with the data collected by the global PATRO Adults study [26]. The results of this analysis are consistent with the latest data of the overall PATRO Adults study, which was analysed in September 2015 (data on file). In the 954 patients recruited so far [mean (SD) age 50.2 (15.2) years; mean (SD) BMI 29.4 (6.1) kg/m^2^], 515 (54%) have been pre-treated with another rhGH. Overall, 1497 AEs have been reported in 473 (49.6%) patients; 245 of these were considered serious. There were 110 ADRs in 68 patients, including 19 general disorders/administration site conditions, 18 nervous system disorders, 16 musculoskeletal/connective tissue disorders and two events of increased IGF-1 levels. A total of 23 SAEs in 16 patients were suspected to be related to treatments, including one case each of: worsening of diabetes mellitus, dyspnoea and multiple endocrine neoplasia type 1. Of the 105 patients who discontinued treatment, 23 (21.9%) patients discontinued due to an AE.

In conclusion, this preliminary analysis shows that, as with other rhGHs, treatment with Omnitrope^®^ is well tolerated in adult patients with GHD in routine clinical practice, with no confirmed relationship with the development of diabetes, no occurrence of malignancy or other additional safety issues. This analysis also confirms the effectiveness of Omnitrope^®^ for the treatment of adults with GHD.
